# Analysis of Mandatory Post‐Marketing Surveillance Studies Supports Revised Regulatory Requirements in Korea

**DOI:** 10.1002/pds.70382

**Published:** 2026-04-27

**Authors:** Kevin Wolter, Seung‐Hee Jeong, Ju‐Won Woo, Eunsol Kim, Yoosun Lee, Sera Cha, Evelyn Moy, Rod Junor

**Affiliations:** ^1^ Internal Medicine Pfizer Research and Development Groton Connecticut USA; ^2^ Real World Evidence Generation Pfizer Research and Development Seoul Korea; ^3^ Portfolio Management Pfizer Research and Development New York New York USA; ^4^ Internal Medicine Pfizer Research and Development Surrey UK

**Keywords:** Korea, MFDS requirements, post‐authorization safety studies, post‐marketing surveillance studies

## Abstract

**Purpose:**

The objective of this analysis was to determine the contribution to the understanding of product benefit/risk made by non‐randomized, observational post‐marketing surveillance (PMS) studies previously required by the Ministry of Food and Drug Safety (MFDS) re‐examination system and conducted by Pfizer in Korea over 2000–2024.

**Methods:**

A retrospective analysis of all Pfizer Korea PMS studies during 2000–2024 was performed, covering a wide range of therapy areas. Available Pfizer clinical study reports and re‐examination reports were reviewed for sample sizes, study periods, and key safety and effectiveness findings.

**Results:**

Twenty‐four studies that enrolled 21 179 Korean participants were identified. Original sample sizes assigned by MFDS were typically either 600 or 3000 participants, but enrollment challenges were common and approximately half of the PMS studies were permitted to reduce sample size. Mean study duration (FSFV–LSLV) was 3.8 years. Safety findings were consistent with existing global safety data, with no clinically significant new safety information being generated from the PMS studies.

**Conclusions:**

PMS conducted by Pfizer in Korea were often challenging to recruit and generated data that were consistent with prior global data, with no evidence of clinically meaningful differences in Korean patients. These findings support MFDS's recent transition to a risk management plan (RMP) framework, with PMS only as needed, reducing mandatory requirements for newly approved drug products. Furthermore, when a PMS study is necessary to address a specific scientific uncertainty, MFDS allows individual determination of the sample size, which will improve the feasibility of conducting the study.

## Key Points

- Retrospective analysis of 24 post‐marketing surveillance (PMS) studies in Korea 2000–2024, involving 21 179 participants to determine their contribution to understanding benefit–risk of medicinal products.
- Most studies were completed within the re‐examination period but 29% required extension due to enrollment difficulties. 50% of PMS required sample size reduction.
- No new safety or effectiveness findings warranted changes to the product label.
- For all products the re‐examination outcome was Approval, with labels updated only to add a brief summary of study‐specific results. No new safety concerns from any PMS warranted new limitations of use, new contraindications/warnings, or new risk minimization measures (RMMs).

## Plain Language Summary

Until 2025, a re‐examination procedure for each new drug was scheduled by the Korean Ministry of Food and Drug Safety (MFDS) to determine if new information existed that needed to be included in the product label. The procedure took place 4 or 6 years after approval and included a mandatory observational post‐marketing surveillance (PMS) study, completed by the Sponsor within that re‐examination period. The present study analyzed all Pfizer PMS studies in Korea during 2000–2024 to determine if they had generated important new safety or effectiveness information. Most of the 24 Pfizer PMS studies in Korea were completed within the assigned re‐examination period (4 or 6 years) while some required an extension (range +1.4 to +3.3 years) due to enrollment difficulties. No effectiveness findings were identified that warranted changes to the product information. For all studies, safety findings were consistent with the current global safety data for the product. No significant new safety risks were identified by any study. It was concluded that these findings lend support to the decision by MFDS to discontinue the previous re‐examination system with mandatory PMS study in favor of a more flexible approach to safety monitoring based on the risk management plan.

## 1 Purpose

Sponsors of a new drug product or a new indication for a marketed product may be required, as a condition of approval, to conduct a post‐marketing study when a regulatory authority has a specific outstanding scientific question related to the new use. In the US and Europe, these include post‐marketing surveillance (PMS) studies, post‐marketing requirements (PMR), Pediatric Research Equity Act (PREA) studies, post‐authorization safety studies (PASS), and post‐authorization efficacy studies (PAES) [1, 2, 3, 4]. In general, these studies seek to generate data that represents “missing information” for the drug, such as safety or effectiveness in populations not studied in the pre‐approval clinical trials, including particular disease severities or subtypes, certain comorbidities, or pediatric patients. While studies with focused scientific objectives can yield important new information, prospective post‐marketing studies can be difficult to enroll, and alternative approaches have been proposed [5].

Starting in 1995, the Korean MFDS established the re‐examination system, which scheduled a formal re‐assessment procedure to occur 4 to 6 years after approval that reviewed new information arising from general use of the drug and required a mandatory prospective, observational study to be completed within that re‐examination period. The study typically had an enrollment target of 600 or 3000 Korean participants, depending on the drug's approval status and/or duration of use in other countries, but independent of the number of potential study participants. Some studies also involved an element of retrospective data capture, for example, from the participant's hospital record.

PMS studies enrolled a general population of Korean patients using the drug for the approved indication under routine care; therefore, while recruitment was not restricted to specific sub‐populations, such participants were not excluded. All adverse events were captured on structured case report forms and MedDRA coded with the objective of identifying new safety risks (e.g., unexpected adverse drug reactions) that might need to be added to the product label. Effectiveness data were captured using pre‐specified endpoints relevant to routine clinical practice.

Before the end of the re‐examination period, the Sponsor submitted a re‐examination report (RER) containing PMS results and other available safety information. The outcome of the re‐examination was communicated by official MFDS letter, and a brief summary of PMS results was typically added to the product label. Although less burdensome than randomized controlled trials (RCTs), PMS still required significant resources and had limitations. For example, as observational studies with no comparator arm, formal comparisons to RCT results were not performed. Instead, PMS findings were qualitatively interpreted in the context of existing evidence.

Beginning in 2015, MFDS began a transition to a unified Risk Management Plan (RMP) framework for post‐marketing safety monitoring, which progressively replaced the re‐examination system that was abolished in February 2025, eliminating the mandatory requirement for a PMS study. Under the RMP framework, drug safety is managed in a comprehensive, lifecycle‐based manner that allows different approaches to active surveillance, depending on the circumstances (e.g., a new indication for an established drug versus a new drug product). When a PMS study is needed to investigate a specific scientific concern, as a part of the RMP, MFDS now allows the Sponsor to propose a suitable sample size considering disease prevalence, market penetration and other factors [6, 7, 8].

Pfizer has a relatively large portfolio of products in Korea and has accumulated many years of experience conducting PMS studies. Given the new direction taken by MFDS in 2025, we performed a retrospective analysis of Pfizer PMS studies in Korea from 2000 to 2024 to determine if these mandatory studies had made an important contribution to our understanding of drug product benefit/risk.

## 2 Methods

A retrospective analysis was performed of all PMS in Korea conducted by Pfizer during 2000–2024. The method for selecting studies from the Pfizer corporate clinical trial registry (CCTR) is similar to that described by Wolter, et al.^6^ for analyzing PMS in Japan. From the set of all clinical studies that consented and enrolled participants in Korea, all interventional clinical trials were excluded, along with studies having planned, ongoing, or cancelled status, as these would have no results available. Finally, studies with first subject/first visit (FSFV) prior to 01‐January‐2000 were excluded, as were studies with sites outside of Korea, since foreign sites were not used in Korean PMS. Protocol titles for the remaining studies were reviewed to confirm the study as a PMS required by MFDS.

To conduct the analysis, study level results were sought from the RER in Korean, which provided a detailed record of the methods and all results including comprehensive information on safety findings, equivalent to a standard clinical study report (CSR) [9]. The CSR for these Korean PMS studies was an English translation of the RER content that was directly pertinent to the study, as an RER also included other content such as routine pharmacovigilance data. The English CSR was used for analysis if the original Korean RER was not available.

Data were analyzed descriptively. Results for safety and effectiveness are based on the overall study conclusion from the study report, not on reanalysis of study data. All adverse events were reported using structured data collection forms. The overall safety conclusion for each study was taken directly from the text of the conclusion section of the study report and was assigned a category. Priority would be given to any conclusion indicating the safety findings of the PMS suggested that use of the drug may entail more risk than was previously known. The next category was a conclusion statement that directly compared the observed study results to the known safety profile for the drug, followed by a category for a conclusion statement that inferred such a comparison, such as “No new safety concerns” or “Safe for approved indications”. In the absence of any comparative safety conclusion, stated or implied, a non‐comparative statement from the report would be reported such as “Well tolerated.”

Since PMS are conducted primarily for safety purposes, not all studies included effectiveness measures but whenever available, overall conclusions regarding effectiveness were tabulated. Re‐examination outcomes, communicated by MFDS via official letter, were obtained from corporate records.

## 3 Results

**FIGURE 1:**
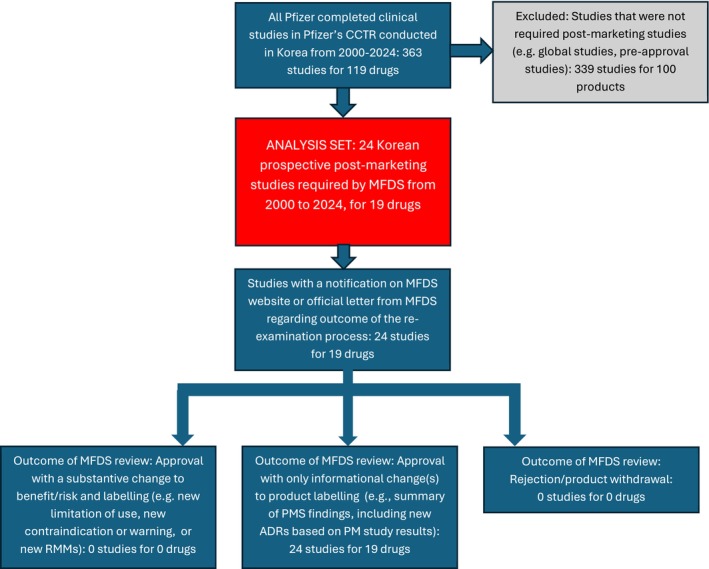
Derivation of the analysis set of 24 Korean MFDS‐required post‐approval studies and outcomes of the re‐examinations.

Between 2000 and 2024, Pfizer conducted 363 interventional and observational clinical studies in Korea. Of these, 24 were prospective, non‐comparative observational studies required by MFDS under the re‐examination system. The 24 studies were for 19 products and enrolled a total of 21 179 participants. The selection of studies included in the analysis set is shown in Figure 1. The therapeutic areas represented by these studies are shown in Table 1.

**TABLE 1:** Therapeutic areas for 24 post‐approval required studies.

Therapeutic area	Number of studies	Sample sizes (% enrollment target achieved)	Sample size reduced by MFDS (*n* of *N*)
Anti‐infectives	2	3000 (8%); 3000 (108%)	1 of 2
Gastrointestinal	1	600 (18%)	1 of 1
Genitourinary	1	3000 (104%)	0 of 1
Immunology	1	600 (35%)	1 of 1
Inflammation	4	3000 (35%); 600 (79%); 600 (87%); 600 (1%)	1 of 4*
Men/Women's Health	3	600 (60%); 3000 (114%); 600 (107%)	1 of 3
Neurology/pain	3	3000 (124%); 600 (117%); 600 (110%)	0 of 3
Oncology	4	3000 (4%); 3000 (7%); 600 (103%); 160 (67%)	3 of 4
Rare diseases	3	600 (30%); 600 (7%); 600 (18%)	3 of 3
Vaccines	2	600 (108%); 600 (110%)	0 of 2
Total	24		11 of 24

Abbreviation: MFDS, Ministry of Food & Drug Safety (Korea).

* Sample size was formally reduced for 1 study, however 3 other under‐enrolling studies (same drug) were allowed to be pooled and reported as a single study.

### 3.1 Sample Size

Original sample sizes, determined by the drug's approval status and usage outside of Korea but not by factors related to patient availability, were set at either 600 (15 of 24 PMS) or 3000 participants (8 of 24 PMS) except for 1 PMS (designated orphan drug) with a target of 160 participants (Table 1). Ten (10) of 24 PMS met the original target while 14 PMS did not, with 11 of 14 having been granted 1 or more successive sample size reductions and MFDS ultimately accepting the number of enrolled participants as reflected in Table 1. The 3 remaining studies unable to achieve their original targets (1 product; 3 indications) were, with MFDS permission, pooled and reported as a single study without individual sample size reductions.

Mean enrollment (% target) did not differ based on the original target number: *N* = 3000, 62%; *N* = 600, 66%; *N* = 160, 67%. Of the 14 studies unable to reach their assigned enrollment target, the mean final enrollment (% original target) was 33%. Differences were noted according to therapeutic area: neurology/pain and vaccine studies all enrolled to original target, while no rare disease or inflammation studies did so. Other therapeutic areas with > 1 study showed mixed results.

### 3.2 Study Duration and Timing

Table 2 shows the duration of each study in the analysis set, along with the timing of the study in relation to the re‐examination period assigned by MFDS, which could be set at either 4 or 6 years. Mean study duration (first subject/first visit to last subject/last visit; FSFV to LSLV) was 3.8 years (median 3.6 years; range 0.5 to 7.8 years). Seventeen (17) of 24 studies (71%) were completed as planned, prior to the end of the assigned re‐examination period. The remaining 7 studies (29%) were completed a mean of 2.3 years beyond the original re‐examination period (range +1.4 to +3.3 years) due to enrollment difficulties; in each case, MFDS agreed to extend the original re‐examination period without penalty.

**TABLE 2:** Duration and time interval relationships for product approval, reimbursement, re‐examination and post‐marketing study conduct.

Study ID (disease area)	NCT ID	Re‐exam Period (yrs.)^a^	Approval to FSFV (months)	Duration (LSLV‐FSFV) (yrs.)	On‐time completion? (overrun)	Re‐exam period extended?	NDA approval date	Approval to Reimbursement (months)	Reimbursement to FAP (months)	Reimbursement to FSFV (months)
A8851025 (AI)	NCT00802854	6	45.1	4.2	No (+2.0 years)	Yes	30‐May‐08	26	−22	19
B1811040 (AI)	NCT01072539	6	33.7	4.9	No (+1.7 years)	Yes	30‐Jul‐07	16	4.2	18
A3921343 (GI)	NCT04071405	4	19.7	2.4	Yes	No	20‐Sep‐18	7	−1.3	12
A0221075 (GU)	NCT00879398	6	13.1	4.8	Yes	No	2‐Oct‐08	10	−4.5	3
B1741015 (Immun)	NCT00484094	6	63.3	4	No (+3.3 years)	Yes	25‐Mar‐06	54	−44	9
A3921249 (Inflamm)	NCT02984020	6	25.4	6.1	No (+2.2 years)	Yes	2‐Apr‐14	11	−4	14
B1801105 (Inflamm)^b^	NCT00195403	6	7	3.8	Yes	No	6‐Oct‐03	7	−5.2	0
B1801112 (Inflamm)^b^	NCT00195416	6	19.9	2.7	Yes	No	6‐Oct‐03	7	13	13
B1801113 (Inflamm)^b^	NCT00195403	6	37	1.2	Yes	No	6‐Oct‐03	7	22	30
A3051083 (Neuro/Pain)	NCT00483002	6	2.9	3.9	Yes	No	30‐Mar‐07	NA	NA	NA
B2061143 (Neuro/Pain)	NCT02548949	6	26.6	3.8	Yes	No	6‐Feb‐14	13	−1.3	14
C0801038 (Neuro/Pain)	NA	6	11.7	2.5	Yes	No	4‐Jun‐10	NA	NA	NA

Study ID (disease area)	NCT ID	Re‐exam Period (yrs.)^a^	Approval to FSFV (months)	Duration (LSLV‐FSFV) (yrs.)	On‐time completion? (overrun)	Re‐exam period extended?	NDA approval date	Approval to Reimbursement (months)	Reimbursement to FAP (months)	Reimbursement to FSFV (months)
A4061075 (Oncology)	NCT02156895	6	70.9	3.1	No (+3 years)	Yes	22‐Aug‐12	70	−70	1
A5991089 (Oncology)	NCT01047358	6	22.3	4.1	Yes	No	24‐Jul‐08	10	−7.5	12
A6181146 (Oncology)	NCT00444795	6	11.2	7.8	No (+2.7 years)	Yes	19‐Jun‐06	8	−0.8	3
B1931027 (Oncology)	NCT04307134	6	18.2	4.5	Yes	No	3‐Jan‐19	9	−30	9
B1821005 (Rare Dis)	NCT00484185	6	14.5	4.8	Yes	No	15‐Jun‐06	3	4.5	12
B1831078 (Rare Dis)	NCT01790828	4	42.3	0.5	Yes	No	11‐Aug‐10	28	0.4	15
B1831086 (Rare Dis)	NCT03034044	4	33.6	1	Yes	No	31‐Mar‐14	6	1.8	28
A6791036 (W/Health)	NCT02104557	6	12.5	6.3	No (+1.4 years)	Yes	29‐Jan‐13	NA	NA	NA
B1781047 (W/Health)	NCT01793142	6	23.3	3.6	Yes	No	16‐Nov‐11	10	−1.2	14
B2311067 (W/Health)	NCT02792504	6	20.2	4.3	Yes	No	25‐Jul‐14	NA	NA	NA
B1851057 (Vaccines)	NCT01509105	6	14.9	4.6	Yes	No	19‐Mar‐10	NA	NA	NA
B1851143 (Vaccines)	NCT01834222	4	18.9	2.3	Yes	No	25‐May‐12	NA	NA	NA

Abbreviations: AI, anti‐infectives; FAP, date of final approved protocol; FSFV, first subject/first visit for a study; GI, gastro‐intestinal diseases; GU, genitourinary diseases; Immun, immunology; Inflamm, inflammatory diseases; LSLV, last subject/last visit for a study; NA, not available/applicable; NCT ID, national clinical trial registration number; NDA, new drug approval; Neuro/pain, neurological diseases and pain control; W/Health, women's health.

^a^ The original duration for the re‐examination period, as assigned by MFDS.

^b^ These 3 studies were pooled and reported as one study.

### 3.3 Relationship of Reimbursement to Study Start‐Up

Table 2 also shows the reimbursement date for drugs granted reimbursement by the governmental National Health Insurance agency, which occurred separately to the PMS study and the re‐examination process. Four (4) products (4 studies) did not receive reimbursement, while 1 vaccine product (2 studies) was provided through the National Immunization Program; these studies do not have a reimbursement date. Therefore, 18 studies (for 15 products) can be assessed for the impact of reimbursement on study operational performance/timing.

Across these 18 studies, reimbursement occurred a mean of 17 months after NDA approval (median: 10 months; range: 3 to 70 months).

The protocol received final approval (FAP) within Pfizer prior to reimbursement being granted for 12 of 18 studies (67%). For these studies, FAP occurred a mean of 16 months prior to reimbursement (median: −4.8 months; range: −0.8 to −69.9 months). The protocol received final approval after reimbursement for 6 of 18 studies (33%). For these studies, FAP occurred a mean of 7.6 months after reimbursement (median: +4.3 months; range: +0.4 to +21.8 months).

Across the 18 studies, the mean time between reimbursement and FSFV was 13 months (median: 13 months; range: 0 to 30 months). Nine (9) of the 18 studies (50%) had a reimbursement‐FSFV interval of 12 months or less; 4 studies (22%) had a reimbursement‐FSFV interval of 18 months or more; for the remaining studies, FSFV occurred between 12 and 18 months after reimbursement was granted.

### 3.4 Effectiveness

Effectiveness was evaluated in 22 of 24 studies; effectiveness was not a study objective for the 2 vaccine studies. The quantitative values reported for effectiveness (data not shown) varied widely across different indications and different therapeutic areas, as would be expected; however, in no case was the measured effectiveness in the observational post‐marketing study judged to be significantly misaligned with the efficacy seen in the corresponding Phase 3 RCTs.

### 3.5 Safety

Table 3 shows the reported incidences for adverse events (AEs) and serious adverse events (SAEs) without regard to a causal relationship between the drug and the event; adverse drug reactions (ADRs) and serious ADRs, for which a causal relationship is suspected; unexpected ADRs (ADRs not listed in the approved product label) and suspected unexpected serious adverse reactions (SUSARs). Since all PMS were non‐comparative observational studies, no comparative conclusion can be drawn.

**TABLE 3:** Safety results by study.

Study identifier (Therapeutic area)	% with any AE	% with any SAE	% with any ADR	% with any serious ADR	% with any unexpected ADR	% with any SUSAR	Overall safety conclusion
A0221075 (GU)	8.5%	0.0%	8.2%	0%	0.1%	0%	Consistent with known profile
A3051083 (Neuro/Pain)	9.3%	0.1%	8.5%	0.05%	0.1%	0%	Well tolerated
A3921249 (Inflamm)	26%	4%	15%	2.0%	3.1%	0.1%	No significant new information
A3921343 (GI)	40%	7.5%	30%	0.9%	12%	0%	No significant new information
A4061075 (Oncology)	83%	13%	71%	6.3%	14%	0%	Consistent with known profile
A5991089 (Oncology)	25%	1.0%	7.3%	0%	3.9%	0%	Well tolerated
A6181146 (Oncology)	81%	12%	77%	7.5%	35%	2.7%	Consistent with known profile
A6791036 (W/Health)	17%	0.4%	23%	0%	0.3%	0%	Consistent with known profile
A8851025 (AI)	58%	42%	3.3%	0%	1.6%	0%	Consistent with known profile
B1741015 (Immun)	54%	7.7%	43%	2.9%	1.0%	2.9%	Consistent with known profile
B1781047 (W/Health)	6.1%	0.5%	3.9%	0.1%	2.7%	0.1%	Consistent with known profile
B1801105, B1801112, B1801113 (Inflamm)*	14%	0.3%	13%	Not reported	2.2%	0%	Safe for approved indications
B1811040 (AI)	33%	13%	9.9%	0.1%	0.4%	0.1%	Consistent with known profile
B1821005 (Rare Disease)	5.1%	2.3%	1.1%	0.6%	0.6%	0%	Consistent with known profile
B1831078 (Rare Disease)	24%	0%	14%	0%	12%	0%	No new safety concerns
B1831086 (Rare Disease)	14%	0%	1.9%	0%	1.0%	0%	Consistent with known profile
B1851057 (Vaccines)	26%	0.5%	13%	0%	4%	0%	No new safety concerns
B1851143 (Vaccines)	21%	0.3%	20%	0%	1.5%	0%	No new safety concerns
B1931027 (Oncology)	93%*	32%	50%	3.7%	14%	0.9%	No new safety concerns
B2061143 (Neuro/Pain)	11%	0.4%	9.1%	Not reported	2%	0%	Consistent with known profile
B2311067 (W/Health)	11%	0.3%	9.1%	0%	1.1%	0%	Consistent with known profile
C0801038 (Neuro/Pain)	27%	1.5%	3.3%	0.5%	0.2%	0%	No new safety concerns

* Safety findings for these 3 studies were pooled for submission to MFDS.

Abbreviations: ADR, adverse drug (related) event; AE, adverse event; AI, anti‐infectives; GI, gastro‐intestinal diseases; GU, genitourinary diseases; Immun, immunology; Inflamm, inflammatory diseases; Neuro/pain, neurological diseases and pain control; SAE, serious adverse event; SUSAR, suspected unexpected serious adverse (drug) reaction–an SAE that may be related to drug but is not currently represented in the drug's reference safety document in terms of its nature, severity or outcome; W/Health, women's health.

The incidence of AEs and SAEs varied widely across the studies, which is consistent with a wide variety of underlying medical conditions being treated. Typically, oncology studies had the highest incidences of adverse events (*n* = 4 studies; range for AE incidence: 25% to 93%; range for SAE incidence: 1% to 32%). By contrast, the incidences of SUSARs were low across all studies (mean = 0.31%; median = 0%; range 0% to 2.9%).

There were 22 overall safety conclusions for the 24 studies since 3 studies were analyzed together. Nineteen (19) conclusions (21 studies) indicated that the safety results of the PMS were consistent with the known safety profile of the product or stated that no “new” safety information was identified in the PMS. For 3 studies, the safety conclusion was less specific and did not refer to the established safety profile (specifically, “well tolerated” or “safe for approved indications”). No PMS study concluded that the risks of the medicinal product may be greater than previously thought.

### 3.6 Re‐Examination Outcomes

Of 22 re‐examination submissions (for 19 products), all were approved by MFDS with a request to provide a brief statement of study results in the product label. No PMS study identified important new safety risks that led MFDS to post the finding on its website or otherwise communicate it broadly.

## 4 Conclusions

PMS studies had been mandatory in Korea for all newly approved drugs. They were believed necessary to compensate for drug development programs with limited geographic scope and for limitations of existing pharmacovigilance systems. However, drug development programs have become geographically broader, with large, more diverse safety populations, and global pharmacovigilance systems are now more effective and impactful.

In February 2025, Korea's transition from a re‐examination system with mandatory PMS to an RMP‐based framework was complete, marking a significant advance in regulatory practice. This transition, led by MFDS, emphasizes proactive safety monitoring and continuous benefit–risk assessment. Increased use of real‐world data for regulatory decision‐making aligns with global standards and enhances scientific robustness. Moreover, to the extent that an RMP‐based framework lessens the need for prospective observational studies, the impact of reimbursement timing on study recruitment will be mitigated.

In light of these changes, our analysis sought to retrospectively assess the contribution PMS studies made to understanding benefit/risk for medicinal products in Korea under the previous re‐examination system.

The strength of this analysis is a large dataset extending over two decades, with data from more than 20 000 participants using 19 medicinal products across a range of therapeutic areas.

The 24 Korean PMS completed by Pfizer during 2000 to 2024 encountered important operational challenges. One factor was the timing of product reimbursement. Until reimbursement is obtained, recruitment is difficult because few patients will have access to the product, which enables study participation. Of 15 products granted reimbursement (18 studies), the mean interval between approval and reimbursement, when accrual could begin in earnest, was 17 months into the assigned re‐examination period. The MFDS‐assigned sample size was also a challenge. Sample size was renegotiated and reduced for 11 of the 24 studies; the mean final enrolment for these 11 studies (as % of original target) was 33%. Of 24 studies, 7 completed after the original re‐examination period had ended (range +1.4 to +3.3 years), requiring an extension. Therefore, MFDS's recent decision to permit evidence‐based sample size calculation for future post‐marketing studies under the RMP framework is a welcome change.

In terms of the value provided by PMS studies, SUSARs (SAEs possibly related to the drug but not mentioned in the product information) are of prime interest. While the ability to detect SUSARs is valuable, only 6 of the 24 studies in this analysis found a SUSAR, and in no instance was the event clinically impactful such that additional risk minimization measures were required for the drug.

Until recently, Japan had a similar re‐examination system to Korea, including mandatory PMS studies. Kanmuri and Narukawa [10] reviewed PMS performed for 150 products in Japan and concluded that routine pharmacovigilance was more effective than PMS studies for identifying new safety risks, including SUSARs. More recently, Wolter et al. [11] reached a similar conclusion regarding PMS studies in Japan. Coinciding with this analysis, PMDA's requirements evolved [12] and, as of 2024, PMS studies are no longer mandatory. In Japan, a required post‐marketing study is assigned only for a specific scientific uncertainty, such as missing information or assessing a specific potential safety risk.

Under the new RMP framework in Korea, global pharmacovigilance is expected to play a larger role in continuously monitoring drug safety, in place of mandatory PMS studies. While obligatory safety reporting in a study is a theoretical advantage, pharmacovigilance involves far larger numbers of patients, which is particularly important for rare events that are unlikely to occur in a PMS study.

Since no PMS studies revealed clinically significant new insights regarding safety or effectiveness, it appears the MFDS review and approval process for new products is robust and can assure acceptable benefit–risk. Overall, the reported safety results from 24 PMS studies were found to align with prior data based on the global populations studied in Phase 3. The analysis of these 24 PMS provided no compelling evidence that Korean patients differed meaningfully from Phase 3 participants regarding safety or effectiveness outcomes, or that the results of mandatory Korean PMS provided important safety insights beyond information available from routine pharmacovigilance, which took place in parallel.

This retrospective analysis had limitations. Importantly, it represents the experience of one Sponsor and may not be generalizable. Also, product labels may have been updated prior to the end of the re‐examination period with new events from routine global pharmacovigilance or other sources. That is, although all products were found to be acceptably labeled for benefit/risk upon re‐examination, this outcome may reflect product labels that had been previously updated through routine pharmacovigilance, unrelated to mandatory PMS studies. Finally, the ability to identify important new safety risks may have been impaired in the 14 studies that under‐enrolled and found no such new risks. However, no important new safety risks were identified in the 10 PMS that achieved or surpassed their original enrollment target.

In Pfizer's experience, the MFDS drug approval process appropriately assures acceptable benefit/risk at the time of approval and mandatory PMS in a general patient population do not meaningfully expand our understanding of product benefit/risk. When genuine scientific uncertainty exists, related to a drug's benefit/risk, a targeted study may be needed to address that uncertainty, but MFDS's decision to no longer require a PMS study for each new approval appears to have been warranted, given the marginal value provided by these studies. As the findings from Pfizer's PMS studies may or may not be generalizable, perspectives from other stakeholders are encouraged.

## Funding

This work was supported by Pfizer.

## Ethics Statement

The authors have nothing to report.

## Conflicts of Interest

The authors declare no conflicts of interest.
